# F138. INVESTIGATING A CAUSAL ASSOCIATION BETWEEN NEUROTICISM AND SCHIZOPHRENIA USING TWO-SAMPLE MENDELIAN RANDOMIZATION

**DOI:** 10.1093/schbul/sby017.669

**Published:** 2018-04-01

**Authors:** Hannah Jones, George Davey Smith, Michael C O’Donovan, Michael J Owen, James Walters, Stanley Zammit

**Affiliations:** 1 University of Bristol; 2 Cardiff University; 3 University of Bristol, Cardiff University

## Abstract

**Background:**

Anxiety is a prominent feature of schizophrenia, present in the prodromal phase of the illness. There is strong evidence that the personality trait neuroticism, an underlying factor strongly associated with anxiety, is genetically correlated with schizophrenia, implying that neuroticism and schizophrenia share genetic risk factors in common. However, a genetic correlation may also suggest a possible role of neuroticism in the pathogenesis of schizophrenia. We therefore performed a Mendelian randomization (MR) analysis using publicly available data to investigate the potential casual association between neuroticism and schizophrenia.

**Methods:**

We performed bi-directional two-sample MR between neuroticism and schizophrenia using the most recent publically available summary-level genome-wide data. Single nucleotide polymorphisms (SNPs) associated with neuroticism (p ≤ 1e-5) and schizophrenia (p ≤ 5e-8) were combined using an inverse-variance-weighted (IVW) multiplicative random effects approach. Impact of potential MR assumption violations were explored using weighted median, weighted mode and MR Egger methods. All analyses were performed using the TwoSampleMR R package.

**Results:**

The IVW MR method provided strong evidence of a casual effect of genetically instrumented neuroticism on risk of schizophrenia (p < 0.001). This causal association was also evident when using the median weighted approach (p = 0.004) but evidence was weaker when using the weighted mode (p = 0.719) and MR Egger approaches (p = 0.439). The MR Egger intercept provided weak evidence of presence of horizontal pleiotropy (p = 0.067), however, the I2GX statistic indicated potential violation of the no measurement error MR assumption. There was also evidence of a causal effect of schizophrenia on neuroticism (IVW p = 0.001, weighted median p = 0.017, weighted mode p = 0.018) however, again, the I2GX statistic indicated potential violation of the no measurement error MR assumption.

**Discussion:**

Assuming certain MR assumptions are met, our results provide evidence of a bi-directional causal association between neuroticism and schizophrenia suggesting a genetic overlap rather than a uni-directional casual association, however, the impact of feedback loops between exposure and outcome cannot be addressed. Although there was evidence of horizontal pleiotropy between neuroticism and schizophrenia, evidence of violation of the no measurement error indicates that the MR Egger results should be interpreted with caution.

